# Supplementary material for: The Small RNA Universe of Capitella teleta
Source: Front Mol Biosci. 2022 Feb 25;9:802814. doi: 10.3389/fmolb.2022.802814 (PMC8915122; doi:10.3389/fmolb.2022.802814)
Supplement: Supplementary file 1 [file DataSheet1.ZIP › Supplement/known/CAPTEscaffold_274_15672.pdf]

Provisional ID	: CAPTEscaffold_274_15672
Score total	: 66159.6
Score for star read(s)	: 3.9
Score for read counts	: 66148.9
Score for mfe	: 2.2
Score for randfold	: 1.6
Score for cons. seed	: 3
Total read count	: 129760
Mature read count	: 129558
Loop read count	: 0
Star read count	: 202

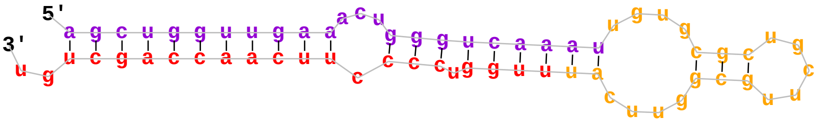

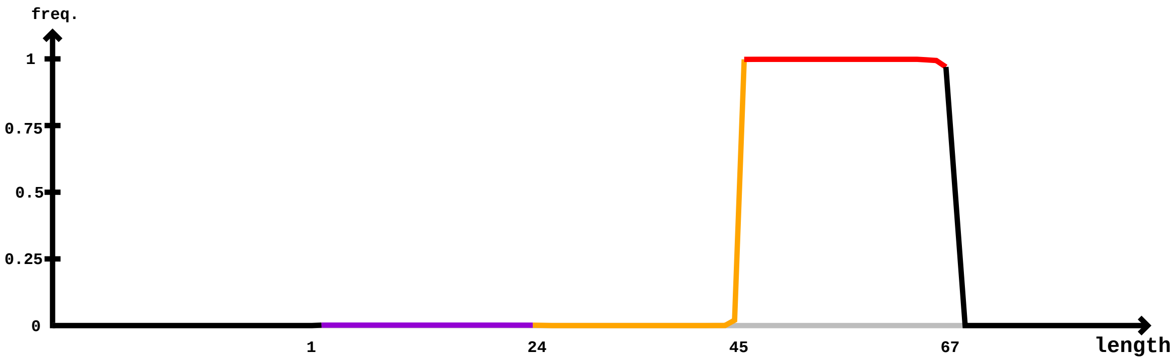

Star Mature

```
5'- cgcagucugaucguagcgcacgccguagcugguugaaacugggucaaauugugcgcugcuugcgguucauuugguccccuucaaccagcugugggugcgcgccacucgcuac   -3'   obs
    cgcagucugaucguagcgcacgccguagcugguugaaacugggucaaauugugcgcugcuugcgguucauuugguccccuucaaccagcugugggugcgcgccacucgcuac         exp
    cgcagucugaucguagcgcacgccguagcugguugaaacugggucaaauugugcgcugcuugcgguucauuugguccccuucaaccagcugugggugcgcgccacucgcuac         known
    .(((((.........((((((.(((((((((((((((...(((((((((....(((.....)))....)))))).))).)))))))))))))))))))))...))).))...    reads   mm   sample

    ......cugaucguagcgcacgccgua.....................................................................................    1       0    seq
    .........................uagcugguugaaacugggucaaa................................................................    1       0    seq
    ..........................agcugguugaaacugggucU..................................................................    1       1    seq
    ..........................agcugguugaaacuggguca..................................................................    1       0    seq
    ..........................agcugguugaaacugggucaa.................................................................    9       0    seq
    ..........................agcugguugaaacugggucaaa................................................................    14      0    seq
    ..........................Ggcugguugaaacugggucaaau...............................................................    5       1    seq
    ..........................agGugguugaaacugggucaaau...............................................................    1       1    seq
    ..........................agcugguugaaacugggucaaaA..............................................................    2       1    seq
    ..........................agcugguugaaacugggucaaUu...............................................................    1       1    seq
    ..........................agcugguugaaacugggucaaau...............................................................    158     0    seq
    ..........................agcuggAugaaacugggucaaau...............................................................    1       1    seq
    ..........................agcugguugaaacugggucGaau...............................................................    1       1    seq
    ..........................agcugguugaaacuAggucaaau...............................................................    1       1    seq
    ..........................agcugguugaaacugAgucaaau...............................................................    1       1    seq
    ..........................agcugguugaaacugggucaaauu..............................................................    5       0    seq
    ...................................................................cauuugguccccuucaaccag........................    3       0    seq
    ...................................................................cauuugguccccuucaaccagc.......................    27      0    seq
    ....................................................................auuugguccccuucaaccagcu......................    1       0    seq
    ....................................................................Cuuugguccccuucaaccagcugu....................    1       1    seq
    .....................................................................uuugguccccuucaacca.........................    1       0    seq
    .....................................................................uuugguccccuucaaccagc.......................    4       0    seq
    .....................................................................uuugguccccuucaaccagcu......................    41      0    seq
    .....................................................................uuugguccccuucaaccagcuU.....................    7       1    seq
    .....................................................................Nuugguccccuucaaccagcug.....................    1       1    seq
    .....................................................................uuugguccccuucaaccagcCg.....................    1       1    seq
    .....................................................................uuugguccccuucaaccagcug.....................    127     0    seq
    .....................................................................uuugguccccuucaaUcagcug.....................    1       1    seq
    .....................................................................Guugguccccuucaaccagcugu....................    1       1    seq
    .....................................................................uuAgguccccuucaaccagcugu....................    2       1    seq
    .....................................................................uAugguccccuucaaccagcugu....................    1       1    seq
```

Star   Mature

```
cgcagucugaucguagcgcacgccguagcugguugaaacugggucaaauugugcgcugcuugcgguucauuugguccccuucaaccagcugugggugcgcgccacucgcuac
.....................................................................uuugguccccuucaacUagcugu.................... 1     1     seq
.....................................................................uuugAuccccuucaaccagcugu.................... 1     1     seq
.....................................................................uuugguccccuucaaccagcuCu.................... 1     1     seq
.....................................................................uuugguccUcuucaaccagcugu.................... 3     1     seq
.....................................................................uuugguUcccuucaaccagcugu.................... 1     1     seq
.....................................................................uuugguAcccuucaaccagcugu.................... 1     1     seq
.....................................................................uuugguccccuucaaccaCcugu.................... 1     1     seq
.....................................................................uuugguccccuucaaccagcAgu.................... 1     1     seq
.....................................................................uuugguccccuCcaaccagcugu.................... 1     1     seq
.....................................................................Nuugguccccuucaaccagcugu.................... 1     1     seq
.....................................................................uuugguccccuucaaccagcugG.................... 3     1     seq
.....................................................................uuuggucGccuucaaccagcugu.................... 1     1     seq
.....................................................................uuugguccccuucGaccagcugu.................... 2     1     seq
.....................................................................Auugguccccuucaaccagcugu.................... 5     1     seq
.....................................................................Cuugguccccuucaaccagcugu.................... 2     1     seq
.....................................................................uuugguccccuucCaccagcugu.................... 1     1     seq
.....................................................................uuuAguccccuucaaccagcugu.................... 1     1     seq
.....................................................................uuuggGccccuucaaccagcugu.................... 1     1     seq
.....................................................................uuugguccccuucaUccagcugu.................... 1     1     seq
.....................................................................uuugguccccuucaaccagcugC.................... 1     1     seq
.....................................................................uuuggucccUuucaaccagcugu.................... 1     1     seq
.....................................................................uuugguccccuucaaccagcugA.................... 4     1     seq
.....................................................................uuugguccccAucaaccagcugu.................... 1     1     seq
.....................................................................uuugguccccuucaaccagcugu.................... 2347  0     seq
.....................................................................uuuggucUccuucaaccagcugu.................... 4     1     seq
.....................................................................uuuggucAccuucaaccagcugu.................... 1     1     seq
.....................................................................uuugguccccuucaaccagcuguU................... 8     1     seq
.....................................................................uuugguccccuucaaccagcuguA................... 8     1     seq
......................................................................uugguccccuucaaccag........................ 2     0     seq
......................................................................uugguAcccuucaaccagc....................... 1     1     seq
......................................................................uugguccccuucaaccagc....................... 237   0     seq
......................................................................uuggucAccuucaaccagc....................... 1     1     seq
......................................................................uuggucUccuucaaccagc....................... 1     1     seq
......................................................................Augguccccuucaaccagcu...................... 1     1     seq
......................................................................uugguccccuucaaccagGu...................... 1     1     seq
......................................................................uuAguccccuucaaccagcu...................... 1     1     seq
......................................................................uugguccccuucaaccagcu...................... 265   0     seq
......................................................................uuCguccccuucaaccagcu...................... 1     1     seq
......................................................................uugguccccuucaacUagcu...................... 1     1     seq
......................................................................Gugguccccuucaaccagcug..................... 1     1     seq
......................................................................uugAuccccuucaaccagcug..................... 1     1     seq
......................................................................uugguccUcuucaaccagcug..................... 4     1     seq
......................................................................uAgguccccuucaaccagcug..................... 2     1     seq
......................................................................uugguccccuucaGccagcug..................... 1     1     seq
......................................................................Augguccccuucaaccagcug..................... 2     1     seq
......................................................................uugguccccuucaaccagcAg..................... 3     1     seq
......................................................................uuggucccUuucaaccagcug..................... 3     1     seq
......................................................................uugguccccuucaaccagcuU..................... 8     1     seq
......................................................................uugguUcccuucaaccagcug..................... 1     1     seq
......................................................................uugguccccuucaacAagcug..................... 1     1     seq
......................................................................uCgguccccuucaaccagcug..................... 1     1     seq
......................................................................uugguccccuucaaccagAug..................... 1     1     seq
......................................................................uuggGccccuucaaccagcug..................... 2     1     seq
......................................................................uugguccccuAcaaccagcug..................... 2     1     seq
......................................................................uugguccccuucaaccagcGg..................... 11    1     seq
......................................................................uugguccccuucGaccagcug..................... 1     1     seq
......................................................................uugguccccuucaaccagcuA..................... 6     1     seq
......................................................................uugguccccuucaaccagcuC..................... 1     1     seq
......................................................................uugguAcccuucaaccagcug..................... 3     1     seq
......................................................................uuggucAccuucaaccagcug..................... 4     1     seq
......................................................................uugguccccuucaaccagcug..................... 2945  0     seq
......................................................................uugguccccCucaaccagcug..................... 1     1     seq
......................................................................Cugguccccuucaaccagcug..................... 1     1     seq
......................................................................uuggucUccuucaaccagcug..................... 2     1     seq
......................................................................uugguccccuucaacUagcug..................... 3     1     seq
......................................................................uugguccccAucaaccagcug..................... 1     1     seq
......................................................................uuggGccccuucaaccagcugu.................... 17    1     seq
......................................................................uugguccccuucaaccagcugG.................... 123   1     seq
......................................................................uugguccccuuAaaccagcugu.................... 32    1     seq
......................................................................uugguccccuucaaccagcGgu.................... 6     1     seq
```

Star Mature

cgcagucugaucguagcgcacgccguagcugguugaaacugggucaaauugugcgcugcuugcgguucauuugguccccuucaaccagcugugggugcgcgccacucgcuac			
....uugguccccCucaaccagcugu....................	24	1	seq
....uugguccAcuucaaccagcugu....................	29	1	seq
....uugguccccuucaaccUgcugu....................	7	1	seq
....uNgguccccuucaaccagcugu....................	6	1	seq
....uugguccccuucaacAagcugu....................	22	1	seq
....uugguccccuucaaccagcuCu....................	15	1	seq
....uugguccccuuGaaccagcugu....................	9	1	seq
....uuggCccccuucaaccagcugu....................	18	1	seq
....uugguccccuucUaccagcugu....................	6	1	seq
....Augguccccuucaaccagcugu....................	229	1	seq
....uGgguccccuucaaccagcugu....................	5	1	seq
....uuggucAccuucaaccagcugu....................	120	1	seq
....uugguccccNucaaccagcugu....................	1	1	seq
....uuAguccccuucaaccagcugu....................	184	1	seq
....uugguccccuucaaAcagcugu....................	18	1	seq
....uugguUcccuucaaccagcugu....................	23	1	seq
....uugguccccuCcaaccagcugu....................	14	1	seq
....uugguccccuucaCccagcugu....................	19	1	seq
....uugguccccuucaacGagcugu....................	19	1	seq
....uugguccccuucaaccaAcugu....................	12	1	seq
....uugguNcccuucaaccagcugu....................	2	1	seq
....uugUuccccuucaaccagcugu....................	7	1	seq
....uugguccccuucaacUagcugu....................	106	1	seq
....uugguccccuucaaccagcuAu....................	18	1	seq
....uuggAccccuucaaccagcugu....................	43	1	seq
....uugguccccuucaaccagcugC....................	15	1	seq
....uuNguccccuucaaccagcugu....................	2	1	seq
....uCgguccccuucaaccagcugu....................	14	1	seq
....uAgguccccuucaaccagcugu....................	48	1	seq
....uugguccccuucaaccGgcugu....................	16	1	seq
....uuggucccAuucaaccagcugu....................	20	1	seq
....Cugguccccuucaaccagcugu....................	11	1	seq
....uugguccccuucaaccagcugA....................	78	1	seq
....uugguccccuucaaUcagcugu....................	34	1	seq
....uugguccccuucaaccagAugu....................	17	1	seq
....uugguccccuucGaccagcugu....................	38	1	seq
....uugguccccuucaaccagcAgu....................	25	1	seq
....uuggucUccuucaaccagcugu....................	106	1	seq
....uugCuccccuucaaccagcugu....................	3	1	seq
....uugguccccuucCaccagcugu....................	2	1	seq
....uuggucGccuucaaccagcugu....................	18	1	seq
....uugguccccAucaaccagcugu....................	46	1	seq
....uugguccccuucaUccagcugu....................	16	1	seq
....uugguccccuucaaccaUcugu....................	12	1	seq
....uugguccccuucaaccagcugu....................	120138	0	seq
....Nugguccccuucaaccagcugu....................	41	1	seq
....uugguccccuucaaccagcuUu....................	9	1	seq
....uuUguccccuucaaccagcugu....................	11	1	seq
....uugguccccGucaaccagcugu....................	3	1	seq
....uugguccccuucaGccagcugu....................	24	1	seq
....uugguAcccuucaaccagcugu....................	25	1	seq
....uugguccccuucaaccagUugu....................	6	1	seq
....uugguccccuuUaaccagcugu....................	31	1	seq
....uuggucccGuucaaccagcugu....................	8	1	seq
....uuCguccccuucaaccagcugu....................	6	1	seq
....uugguccccuucaaccagcCgu....................	12	1	seq
....uugguccccuAcaaccagcugu....................	49	1	seq
....uugguccccuGcaaccagcugu....................	6	1	seq
....uugguccccuucaaccaCcugu....................	2	1	seq
....uugguccccuucaaccCgcugu....................	1	1	seq
....uugguccccuucaaccagGugu....................	6	1	seq
....uugAuccccuucaaccagcugu....................	24	1	seq
....uugguGcccuucaaccagcugu....................	7	1	seq
....uugguccGcuucaaccagcugu....................	5	1	seq
....uugguccccuucaaGcagcugu....................	7	1	seq
....uugguccUcuucaaccagcugu....................	80	1	seq
....Gugguccccuucaaccagcugu....................	29	1	seq
....uuggucccUuucaaccagcugu....................	86	1	seq
....uugguccccuucaaccagcuguU...................	610	1	seq
....uugguccccuucaaccagcuguC...................	5	1	seq

```
                          Star                                          Mature
cgcagucugaucguagcgcacgccguagcugguugaaacugggucaaauugugcgcugcuugcgguucauuugguccccuucaaccagcugugggugcgcgccacucgcuac
......................................................................uugguccccuucaaccagcuguA...................    506    1    seq
.......................................................................ugguccccuucaaccagc.......................    1      0    seq
.......................................................................ugguccccuucaaccagcug.....................    1      0    seq
.......................................................................uUguccccuucaaccagcugu....................    5      1    seq
.......................................................................ugguccccuucaaccagcugu....................    29     0    seq
........................................................................gguccccuucaaAcagcugu....................    1      1    seq
........................................................................gguccccuucaaccagcugu....................    6      0    seq
.........................................................................guccccuucaaccagcugu....................    1      0    seq
..........................................................................uccccuucaaccagcugu....................    5      0    seq
```
